# Visualizing Protein Localizations in Fixed Cells: Caveats and the Underlying Mechanisms

**DOI:** 10.1021/acs.jpcb.3c01658

**Published:** 2023-05-10

**Authors:** Shawn R. Yoshida, Barun K. Maity, Shasha Chong

**Affiliations:** † Division of Chemistry and Chemical Engineering, California Institute of Technology, Pasadena, California 91125, United States; ‡ Division of Biology and Biological Engineering, California Institute of Technology, Pasadena, California 91125, United States

## Abstract

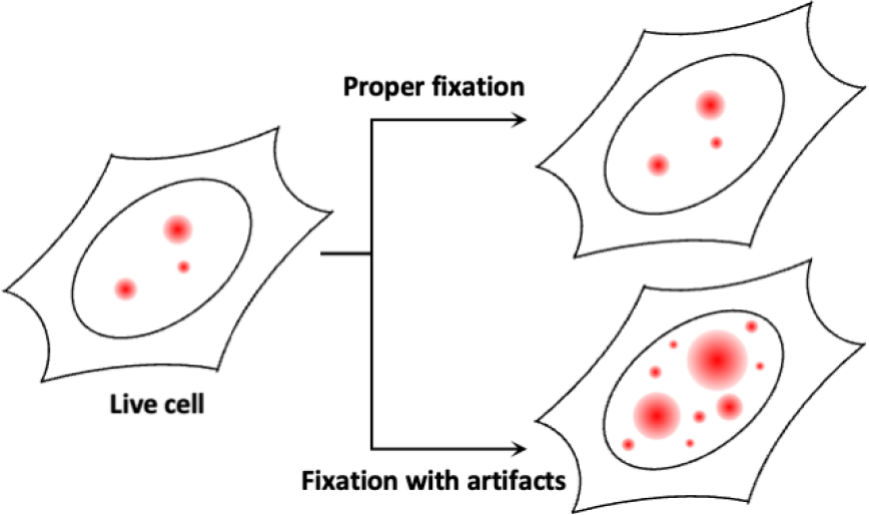

Fluorescence microscopy techniques have been widely adopted in biology for their ability to visualize the structure and dynamics of a wide range of cellular and subcellular processes. The specificity and sensitivity that these techniques afford have made them primary tools in the characterization of protein localizations within cells. Many of the fluorescence microscopy techniques require cells to be fixed via chemical or alternative methods before being imaged. However, some fixation methods have been found to induce the redistribution of particular proteins in the cell, resulting in artifacts in the characterization of protein localizations and functions under physiological conditions. Here, we review the ability of commonly used cell fixation methods to faithfully preserve the localizations of proteins that bind to chromatin, undergo liquid–liquid phase separation (LLPS), and are involved in the formation of various membrane-bound organelles. We also review the mechanisms underlying various fixation artifacts and discuss potential alternative fixation methods to minimize the artifacts while investigating different proteins and cellular structures. Overall, fixed-cell fluorescence microscopy is a very powerful tool in biomedical research; however, each experiment demands the careful selection of an appropriate fixation method to avoid potential artifacts and may benefit from live-cell imaging validation.

## Introduction

Visualizing the subcellular locations of proteins is essential for understanding their functions and regulation. Fluorescence microscopy is among the most popular methods for characterizing protein localizations in the cell, which affords high specificity and sensitivity without requiring complicated sample preparation. Since fluorescence microscopy features an inherent trade-off between spatial and temporal resolution, precisely determining the locations of highly dynamic proteins is often difficult in living cells. Fixation provides an attractive way to preserve snapshots of cells, allowing for high-spatial-resolution measurements of protein localizations that are too dynamic to be characterized in living cells. Although fixation has been thought to faithfully preserve live-cell conditions, an increasing number of works have demonstrated that fixation can sometimes artificially redistribute proteins in the cell, where the artifacts depend on the fixation protocol being used and the protein or cellular structure being studied.^1−8^ While it is possible to accurately determine the subcellular localizations of specific proteins using appropriately chosen fixation methods, there is currently no single method that can perfectly preserve the localizations of all the proteins. When feasible, fixed-cell imaging experiments have been suggested to be supplemented by live-cell imaging to ensure that fixation does not introduce artifacts.^1^

In this Review, we discuss works that characterize the effect of fixation on subcellular protein localizations and develop methods to reduce fixation artifacts. We first briefly introduce three common ways to label a protein in the context of fixed-cell fluorescence microscopy—immunostaining, genome editing to label an endogenous protein, and exogenous expression of a protein fused to a fluorescent tag—along with two widely used classes of fixatives, e.g., cross-linking (aldehydes) and coagulating (organic solvents) fixatives. We then review studies that report fixation-introduced artifacts while imaging a variety of proteins and structures in the cell, including chromatin-binding proteins, liquid–liquid phase separation (LLPS) droplets, membrane receptor clusters, the Golgi apparatus, the endoplasmic reticulum (ER), mitochondria, and ciliary proteins. The qualities of fixation for different proteins are summarized in Table 1. We finally discuss the proposed mechanisms underlying the fixation artifacts and strategies for minimizing the artifacts, explore possible alternative fixation methods, and identify future directions for the development of novel fixatives.

**Table 1:** Summary of the Quality of Fixation in Detecting the Subcellular Localizations of Different Proteins

proteins	fixatives	artifact-free	references
calnexin	4% PFA	yes	(18)
	methanol		
	ethanol		
	2-propanol		
Calreticulin	4% PFA	yes	(18)
	methanol		
	ethanol		
	2-propanol		
CD31, CD44	1% PFA with 0.2% GA	yes	(11)
	4% PFA with 0.2% GA		
CD31, CD44	1% PFA	no	(11)
	4% PFA		
Esrrb	4% PFA	no	(2)
Esrrb	3.7% glyoxal	yes	(34)
Esrrb	2 mM DSG followed by 4% FA	yes	(33, 34)
EWS::FLI1	4% PFA	yes	(1)
FoxA1	3.7% FA	no	(7)
Foxo1, Foxo3a	4% PFA	no	(2)
FUS	4% PFA	yes	(1)
GATA1	0.4% PFA	no	(6)
GOLGB1	4% PFA	yes	(18)
	methanol		
	ethanol		
	2-propanol		
H2B	4% PFA	yes	(4)
	glyoxal		
	4% PFA with 2% GA		
	70% ethanol		
H2B	1% PFA	yes	(2)
HMGB1	4% PFA	no	(27)
HMGB1, HMGB2	methanol/acetone mixture	yes	(5)
HMGB1, HMGB2	4% PFA	no	(5)
HMGN1, HMGN2	4% PFA	no	(5)
HMGN1, HMGN2	2% PFA	no	(28)
HNF1β	100% methanol	yes	(8)
HNF1β	4% FA	no	(8)
Hsf1	4% PFA	yes	(2)
Klf4	4% PFA	no	(2)
LYVE-1	1% PFA with 0.2% GA	yes	(11)
	4% PFA with 0.2% GA		
MeCP2	4% PFA	yes	(3)
Oct4	4% PFA	no	(2)
Sox2	4% PFA	no	(2)
Stat3	4% PFA	yes	(2)
Sp1	4% PFA	no	(2)
TBP	1% PFA	yes	(2)
TOM22	methanol	no	(18)
	ethanol		
	2-propanol		

## Fluorescent Labeling Strategies

**Figure 1:**
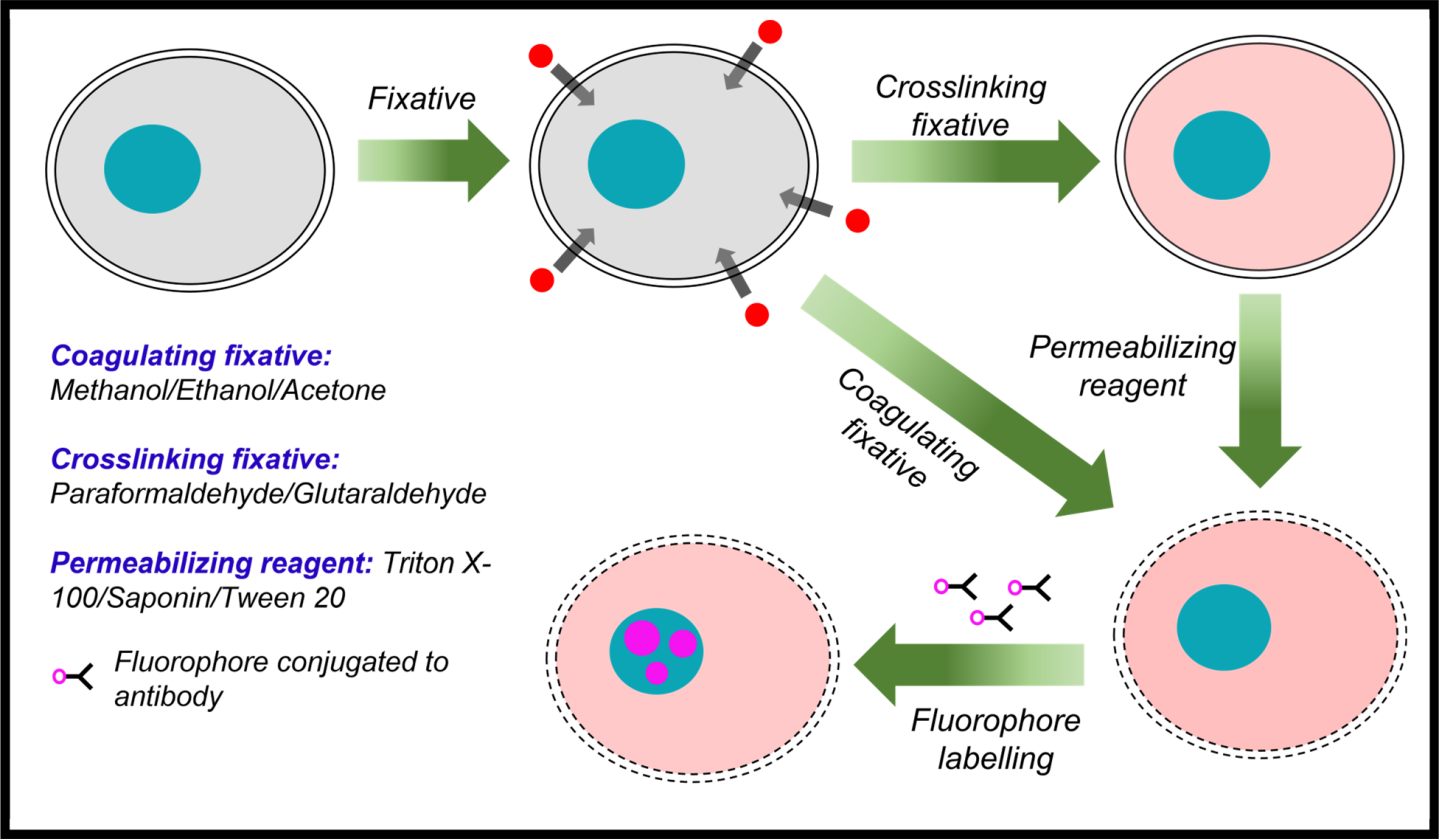
Workflow of immunofluorescence. Cells are first fixed using a cross-linking fixative or fixed and permeabilized using a coagulating fixative. Cross-linking fixatives cannot permeabilize the cell membrane alone, so an additional permeabilization step is required. The fixed and permeabilized sample is then labeled using fluorophores conjugated to antibodies that target the proteins of interest.

Labeling a protein of interest is a critical step of studying the protein using fluorescence microscopy. Among numerous labeling methods, three strategies are broadly applicable to any protein of interest in the cell, including immunostaining, genome editing to label an endogenous protein, and expressing an exogenous protein fused to a fluorescent tag. Immunostaining uses a fluorescently labeled antibody targeting the protein of interest and is, by nature, a fixed-cell technique, as cells must be fixed and permeabilized to allow the antibody to enter the cell and bind to its target protein (Figure 1). While immunofluorescence offers a relatively simple and fast way to image any endogenous protein that has a high-quality antibody available, it requires the careful selection of a fixation/permeabilization protocol that balances fixation efficiency and epitope access. An alternative method of labeling an endogenous protein is to use genome editing techniques, e.g., CRISPR,^9^ to knock-in the cDNA of a fluorescent tag into the genomic sequence that encodes the target protein. Genome editing takes a significantly longer time (usually months) than immunostaining (hours) and requires verification that tagging the endogenous protein does not affect its functions, but in return, enables live-cell imaging of the protein at native expression levels. Cell fixation and permeabilization are not required for visualizing an endogenous protein that is labeled via genome editing, but these treatments can be necessary for more complex experiments, such as simultaneously imaging the protein and other biomolecules, e.g., nucleic acids, through fixed-cell based fluorescence *in situ* hybridization (FISH) methods.^10^ Expressing an exogenous protein fused to a fluorescent tag is another strategy for labeling and imaging the protein in the cell, which demands controls similar to the genome editing-based labeling strategy to guarantee that the protein’s function is unaffected by tagging. The exogenous expression strategy generally takes more time than immunostaining, but less time than genome editing. This strategy sometimes requires selecting cells with exogenous expression levels comparable to the protein’s native expression levels to study protein functions under near physiological conditions. Like the genome editing-based strategy, exogenous expression enables live-cell imaging of proteins of interest, but cell fixation and permeabilization is unavoidable in specific applications, including simultaneously imaging the protein and nucleic acids. In short, while each of the three methods has its own benefits, pitfalls, and unique sets of considerations, they are all susceptible to fixation artifacts.

## Fixation Strategies

Two classes of chemical fixatives are broadly used in fixed-cell fluorescence microscopy: cross-linking fixatives, i.e., aldehydes including paraformaldehyde (PFA) and glutaraldehyde (GA), and coagulating fixatives, i.e., organic solvents including methanol, ethanol, and acetone. Aldehydes react with proximal amino acid side chains to form methylene bridges, cross-linking proteins *in situ*. Upon being dissolved in water, PFA is depolymerized to monomeric formaldehyde (FA) that is practically capable of cross-linking biomolecules. Compared with GA, FA has a smaller molecular weight and thus diffuses throughout the cell more quickly. In contrast, being a dialdehyde consisting of a pentane with formyl groups at C-1 and C-5, the bifunctionality of GA allows it to form inter- and intramolecular covalent cross-links more quickly and over longer distances than FA. PFA and GA are often used in combination to better preserve subcellular localizations of proteins like membrane receptors,^11,12^ tubulins,^13^ and some cytoplasmic proteins.^14^ It is theorized that PFA and GA used together can achieve a more stable fixation output due to their complementary fixation rates and distances.^15,16^ In contrast, coagulating fixatives including methanol, ethanol, and acetone denature and precipitate proteins through rapid dehydration, a process generally faster than cross-linking using aldehydes. However, they are known to extract various biomolecules including lipids and cytosolic and nuclear proteins, and the rounds of dehydration and rehydration can noticeably alter the appearance of many subcellular structures.^17,18^

Immunofluorescence introduces additional considerations, where cell fixation must be followed by permeabilizing cell membranes to allow for antibody access and preserve the epitopes to allow for antibody binding. Cross-linking fixatives themselves do not permeabilize cell membranes; thus, permeabilization is typically performed after cross-linking using detergents, e.g., saponin, Triton X-100, and Tween-20, which remove lipids and cholesterol from the membrane.^17,19,20^ In addition, the structural stability afforded by cross-linking fixatives, or their combination, comes at the cost of restricted access to epitopes and autofluorescence of the aldehydes themselves.^21−23^ In contrast, organic solvents precipitate out lipids throughout the entire cell, fixing the cell and permeabilizing its membranes in one step. They also preserve epitopes well and do not exhibit significant autofluorescence compared with aldehydes.^17,19,20^

Nevertheless, there is currently no universal, artifact-free fixation method that perfectly preserves the appearance of cells. Different proteins and subcellular structures have been found better preserved by different cell fixation protocols. Below, we review the effects of different fixation methods on various systems of interest in the cell and discuss the live-cell experiments used to identify the fixation artifacts.

## Fixing Proteins That Bind to Chromatin

Numerous proteins are associated with chromatin in mammalian cells. Characterizing where and when they bind on chromatin is critical to understanding their functions. For example, during mitosis when transcription is stopped and chromatin condenses, it is thought that mitotic bookmarking, i.e., the association of a small number of critical transcription factors (TFs) to mitotic chromatin, might enable daughter cells to reestablish their intended transcriptional program after mitosis.^2,6,24^ The localizations of chromatin architectural high mobility group (HMG) proteins are also thought to play a role in the regulation of mitotic chromatin.^25,26^ A large body of work used immunofluorescence to show that TFs and HMG proteins appear to be evicted from mitotic chromatin. However, more recent reports suggest that these observations are inconsistent with live-cell fluorescence images and can largely be attributed to fixation artifacts.

Specifically, after fusing high mobility group box (HMGB) and high mobility group N (HMGN) proteins to a fluorescent tag and expressing them in HeLa cells, Pallier et al. found that the proteins bind to chromatin throughout early to late phases of mitosis in live cells,^5^ contrary to previous findings based on immunofluorescence that endogenous HMGB and HMGN proteins dissociate from mitotic chromatin.^27,28^ Consistently, fluorescently labeled HMGB1, HMGB2, HMGN1, and HMGN2 strongly colocalize with Hoechst-labeled condensed chromatin in live mouse 3T3 cells, which become largely excluded from the compacted chromatin upon cell fixation with PFA. The authors verified that the discrepancy was not a result of fluorescent tagging of proteins or their ectopic expression levels. Interestingly, however, fixing cells with a methanol/acetone mixture temporarily preserved the HMGB–chromatin association, though it ultimately caused nearly all fluorescence signal to be lost by the end of the fixation protocol, proving to be an inappropriate fixative. Because the fixation artifact was only observed upon PFA and FA treatments, the authors argued that (para)formaldehyde-based fixation may prevent HMG–chromatin binding by distorting the chromatin structure or the structure of the HMG proteins themselves.

The fixation-induced release of mitotic chromatin-associated proteins is not unique to HMG proteins upon PFA fixation. Instead, this artifact has been found replicated across many TFs upon cell fixation by both cross-linking and coagulating fixatives. Whereas previous works used immunofluorescence to show that most TFs are excluded from chromatin during mitosis,^29−32^ Teves et al. imaged a variety of endogenously expressed Halo-tagged TFs, including SRY-box transcription factor 2 (Sox2), octamer-binding transcription factor 4 (Oct4), estrogen related receptor beta (Esrrb), Krüppel-like factor 4 (Klf4), specific protein 1 (Sp1), forkhead box protein O1 (Foxo1), and forkhead box O3 (Foxo3a), in live mouse embryonic stem cells and showed that they are all enriched at the mitotic chromatin but released into the nucleoplasm upon cell fixation with PFA at different concentrations and methanol. It is noteworthy that among all the TFs investigated in this work, Sox2 is the only one that has an HMG domain, suggesting that the reported fixation artifact is not limited to chromatin-associated proteins with HMG domains. Other works have found similar artifacts where TFs that associate with mitotic chromatin in live cells are evicted upon fixation, including GATA binding protein 1 (GATA1) upon fixation by 0.4% PFA,^6^ forkhead box protein A1 (FoxA1) upon fixation by 3.7% FA,^7^ and hepatocyte nuclear factor 1β (HNF1β) upon fixation by 4% FA.^8^ Notably, in all the studies discussed here, the cross-linking fixatives (various concentrations of PFA and FA) but not the coagulating fixatives all induced similar fixation artifacts where TFs were evicted from mitotic chromatin. While fixation using a methanol/acetone solution temporarily preserve the binding of HMGB1 to mitotic chromatin,^5^ fixation using 100% methanol still resulted in significantly reduced Sox2 enrichment on mitotic chromatin.^2^ Interestingly, cell fixation by both −20 °C and room temperature methanol was shown to completely preserve binding of HNF1β to mitotic chromatin.^8^ The difference in fixation outcomes could be due to the different proteins of interest, the different fixation protocols used, or the combination of the two, yet these findings do not clearly suggest any one coagulative fixation protocol over another.

It is noteworthy that some cross-linking fixatives are reported to preserve the binding of specific proteins to mitotic chromatin. Whereas Teves et al. demonstrated that Esrrb is evicted from mitotic chromatin upon fixation with 4% PFA,^2^ two studies by Festuccia et al. showed that the mitotic chromatin binding activities of Esrrb can be preserved by fixation with 3.7% glyoxal alone and by fixation with 2 mM disuccinimidyl glutarate (DSG) followed by 4% FA.^33,34^ Glyoxal is a bifunctional cross-linker with a faster fixation rate than PFA and it has been increasingly used in recent super-resolution studies.^35^ DSG is also a bifunctional cross-linker and, like GA, is able to cross-link proteins over longer distances than FA alone.^36,37^ While the interesting findings by Festuccia et al. demonstrate the promise of fixation protocols using glyoxal and DSG combined with FA, the fixation quality is possibly dependent on the protein of interest. It is recommended that researchers carefully choose a fixation protocol for a specific chromatin-associated protein and compare against live-cell images if fixed-cell techniques are required to investigate the protein localization.

## Fixing Proteins That Undergo LLPS

Over the past decade, LLPS has garnered much interest in the biology research community as a potential mechanism by which biomolecules may rapidly self-organize in the complex cellular environment. Unlike many membrane-bound structures where biomolecules are contained by a membrane with protein channels that allow specific biomolecules to pass in and out, LLPS is driven by high levels of transient, selective, and multivalent interactions between the intrinsically disordered regions (IDRs) of proteins comprising the droplet.^38,39^ While rigorous characterization of LLPS *in vivo* remains a question under active investigation,^40,41^ detection of discrete puncta that have a spherical shape, undergo fusion and fission, and dynamically exchange biomolecules with the surrounding according to fluorescence recovery after photobleaching (FRAP) is often considered evidence of putative LLPS in living cells. While such diverse measurements have been widely used for studying proteins under overexpression conditions, far fewer approaches are available to probe LLPS under physiological conditions. Detection of local high-concentration regions or puncta of an endogenously expressed protein using immunofluorescence of fixed cells has been used in an increasing number of studies as evidence of LLPS.^42−46^ This assumes that fixation faithfully preserves multivalent interactions and LLPS formed in living cells. Surprisingly, however, our recent study suggested that this is not always true. We expressed in human U2OS cells various fluorescently labeled proteins that are known to undergo LLPS, including the IDRs of fused in sarcoma (FUS), Ewing sarcoma breakpoint region 1/EWS RNA binding protein 1 (EWSR1), and TATA-box binding protein associated factor 15 (TAF15), and we compared the appearance of their LLPS puncta in living cells and after fixation using cross-linking fixatives, e.g., PFA at different concentrations and a combination of PFA and GA. We found that fixation can both enhance and diminish the appearance of LLPS in living cells by significantly changing specific proteins’ puncta sizes and numbers. Although some of our tested proteins, i.e., EWS::FLI1, full-length FUS, and TAF15 fused to ferritin heavy chain (FTH1), do not have their punctate distributions significantly changed upon cell fixation, this work presents a caveat in studying LLPS using fixation-based methods including immunofluorescence and again highlights the need to use live-cell techniques to verify that fixation does not artifactually redistribute proteins with a LLPS potential in the cell.

## Fixation Quality Can Be Related to Protein Binding Dynamics

While it is difficult to know *a priori* how fixation with a given protocol can affect the subcellular localization of a particular protein, an increasing number of reports have shown that proteins undergoing faster binding/unbinding events are more poorly preserved by fixation. Chromatin-associated proteins have provided prominent examples. Schmiedeberg et al. compared wild-type methyl-CpG binding protein 2 (MeCP2), a DNA-binding protein, and mutants with weakened and more transient chromatin interactions. They found that only the wild-type and mutants with chromatin-binding residence half-times longer than 5 s as measured by FRAP were well-preserved by 4% PFA fixation.^3^ Consistently, it has been shown that many stable chromatin-binding proteins are well preserved by fixation, including the histone protein H2B,^2,4^ TATA-binding protein (TBP),^2^ and CCCTC-binding factor (CTCF).^47,48^ In contrast, Sox2, which more transiently binds to mitotic chromatin than H2B according to FRAP and single-particle tracking (SPT) measurements, is artificially released to the nucleoplasm upon fixation with PFA.^2^ These findings collectively suggest that proteins that bind to chromatin with longer residence times are better preserved by fixation. Similarly, we recently reported that proteins have their intracellular LLPS puncta better preserved by fixation if their multivalent protein–protein interactions underlying LLPS are less dynamic. Specifically, we compared Halo-tagged TAF15 and TAF15-FTH1 fusion proteins with both SPT and fixed-cell imaging assays. We found that TAF15 binds to its LLPS puncta with much shorter residence times than TAF15-FTH1. While the puncta of TAF15 have their sizes and number significantly reduced upon PFA fixation, the appearance of LLPS of TAF15-FTH1 was well preserved by fixation with 4% PFA. These results suggest that both chromatin-associated proteins and proteins with a LLPS potential tend to have their intracellular distributions better preserved by fixation if they have slower binding/unbinding rates to interaction partners.

**Figure 2:**
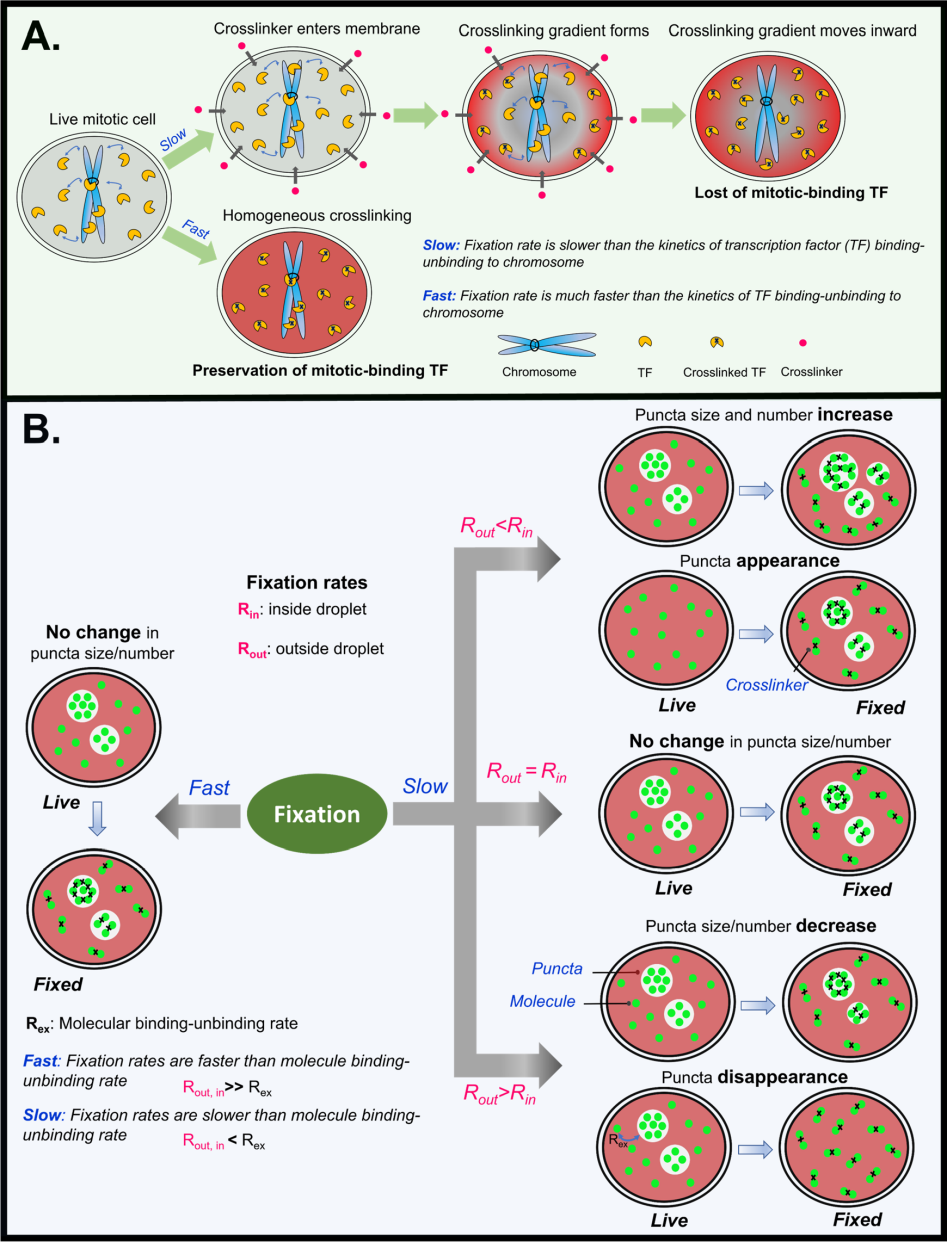
Mechanisms underlying fixation artifacts in cells. (A) Model of cross-linking fixative-caused mislocalization of TFs. TFs dynamically bind and unbind chromatin in mitotic cells. During fixation, the fixative penetrates the cell membrane, and forms a cross-linking gradient toward inside of a cell. Cytoplasmic TFs are cross-linked first, decreasing the population of TFs available to bind to chromatin. This results in reduction of chromosome-bound TFs and introduces a fixation artifact (Slow). However, if the overall rate of fixation (accounting for the diffusion of the fixative and completion of cross-linking) is much faster than the rate of binding and unbinding of TFs to mitotic chromatin, the fixation artifact is minimized (Fast). (B) Model of fixation artifacts for proteins that form puncta, including LLPS droplets, in cells. When the fixation rate is much slower than the binding and unbinding rates of protein molecules to puncta, artifacts are introduced (Slow). Faster in-puncta fixation causes artificial puncta to appear and may increase the size of existing puncta (right top). Slower in-puncta fixation causes puncta to artificially disappear and may decrease the size of existing puncta (right bottom). When the fixation rates in-puncta and out-of-puncta are equal, there is no fixation artifact regardless of the rate of fixation relative to the binding and unbinding rates of molecules to puncta (right middle). There is also no fixation artifact when the fixation rate is much faster than the binding and unbinding rates of molecules to puncta (left).

Understanding the mechanism underlying this trend can help inform potential ways to mitigate fixation artifacts. Teves et al. proposed a model that explains the mislocalization of specific TFs from mitotic chromatin by first noting that these TFs rapidly exchange between chromatin-bound and freely diffusing states and that cross-linking fixation methods rely on the diffusion of aldehydes that permeate the cell from the membrane inward, forming a cross-linking gradient (Figure 2A). Since this cross-linking gradient moves inward, it would initially fix freely diffusing TFs near the periphery of the cell and slowly deplete the pool of TFs available to bind to chromatin. By the time the cross-linking gradient reaches the mitotic chromatin, most TFs would have already been fixed in their freely diffusing states out of chromatin, leaving the mitotic chromatin void of bound TFs. In addition to successfully predicting that more dynamic chromatin binding is more poorly preserved by fixation, this model also predicts that the fixation artifact would increase with the concentration of PFA, which the authors confirmed experimentally by quantifying the chromatin enrichment of Sox2 upon fixation at different concentrations of PFA.

In principle, this model of fixation is applicable not only to TFs dynamically interacting with mitotic chromatin but also generally to proteins that dynamically interact with a centrally located structure in the cell, e.g., LLPS puncta in the nucleus, where we would expect that the cross-linking gradient would fix the freely diffusing protein molecules near the cell periphery first and deplete the puncta as a result. However, this is inconsistent with our observation that the LLPS puncta of specific proteins can increase in number and become larger upon fixation. The discrepancy motivated us to propose a new model of fixation where we take into consideration the dependence of fixation rates on different proteins and their microenvironments, which results in potentially different fixation rates for proteins bound to LLPS puncta and for proteins freely diffusing outside of puncta (Figure 2B). We quantified the fixation-induced change of LLPS appearance by computing the difference of the steady-state percentages of the protein molecules bound to puncta before and after addition of the fixative. Our computations suggest that (1) if the fixation rates of bound and unbound protein molecules differ, molecules will be artificially enriched in the state (bound or unbound) with a faster fixation rate; (2) there will be no artifact if the fixation rates of bound and unbound molecules are identical; and (3) a faster overall fixation rate relative to the dynamics of molecule binding and unbinding will minimize artifacts. We also successfully simulated the bifurcating fixation artifacts on LLPS systems that were observed experimentally and showed that both LLPS-enhancing and diminishing effects are possible depending on the relative fixation rates of the protein in and out of its puncta.^1^ Predictions based on our model are consistent with experimental observations^1−8^ that more dynamic protein-binding events are more poorly preserved by fixation and further suggest that the severity of fixation artifacts is dependent on the overall rates of fixation relative to protein binding, rather than protein binding and unbinding rates themselves. This is consistent with the observation that while PFA artificially evicts Esrrb from mitotic chromatin, glyoxal, a cross-linker with a faster fixation rate than PFA, faithfully preserves this mitotic binding activity.^2,33,34^ Furthermore, our model explains PFA/FA fixation-induced depletion of TFs from mitotic chromatin as that the fixation rate of TF molecules bound to chromatin is slower than that of unbound molecules, which is consistent with the explanation using the cross-linking gradient model.

## Fixation Artifacts Might Happen Regardless of Protein Binding Dynamics

Many cellular proteins besides those bound to chromatin or undergoing LLPS are known to have their distribution affected by fixation. While the balance of fixation and protein binding dynamics are predicted to play a role in these fixation artifacts according to above-discussed models, some of the artifacts are also thought to be caused by mechanisms such as incomplete fixation and disruption to organelle morphology. In addition, some of the reported fixation artifacts remain unexplained, and the underlying mechanisms are under active investigation.

One group of proteins reported to have fixation artifacts are membrane receptors, which are typically dispersed across the cell membrane and cluster in response to stimuli to trigger critical signaling pathways. Because the clustering events are quite transient, characterizing them in live cells can be difficult, making fixed-cell imaging attractive. However, membrane proteins and receptors have been found to artifactually cluster in immunofluorescence images. Specifically, Stanly et al. showed that the transmembrane receptors lymphatic vessel endothelial hyaluronan receptor 1 (LYVE-1), cluster of differentiation 31 (CD31), and cluster of differentiation 44 (CD44) diffusely localize in live cell membranes but artifactually cluster upon fixation with both 1% and 4% PFA and that this postfixation redistribution can be prevented using 0.2% GA in addition to PFA.^11^ According to SPT and FRAP measurements, many membrane molecules including transmembrane proteins and lipids remain mobile upon fixation with only PFA, but not with a combination of PFA and 0.2% GA.^11,12^ In immunofluorescence experiments involving cell fixation by PFA alone, membrane receptors remain largely mobile after incomplete fixation and adding a specific receptor’s secondary antibody causes accretion of the unfixed receptor, resulting in artifactual clustering of the receptor. These findings highlight the importance of live-cell controls for fixed-cell studies, measuring postfixation diffusion in assessing the quality of fixation, and carefully selecting an appropriate fixation protocol, which could vary with the biomolecule of interest.

Artifacts in fixed-cell imaging are also reported to occur due to the physical deformation of organelles or subcellular structures of interest in response to fixation. In search of a single fixation protocol that is suitable for proteome-wide localization studies using immunofluorescence, Stadler et al. compared the effects of 6 fixation protocols, involving both coagulative and cross-linking fixatives, on the localizations of 18 proteins that are associated with different organelles and subcellular structures.^18^ This screening approach provides useful information on which organelles and subcellular structures are and are not well preserved by different fixation protocols. For example, while the localizations of protein markers of endoplasmic reticulum (ER) and Golgi apparatus were robustly preserved by all fixation protocols tested, those of mitochondria were poorly preserved by coagulative fixatives. Specifically, mitochondria in cells appeared to collapse into aggregates near the nucleus after being dehydrated with ethanol and methanol. The authors concluded that fixation via PFA followed by permeabilization via Triton X-100 is generally the best fixation protocol, though it is still not ideal for certain proteins of interest; for example, a combination of FA and Triton X-100 is not suitable for immunostaining of lipid droplet-associated proteins.^49^ Also, the study by Stadler et al. would have benefited from a comparison of the fixed-cell data against the live-cell localizations of the proteins investigated.

Recent work examining the effects of fixation on the localizations of ciliary proteins highlights that different fixation protocols can be required even for proteins associated with the same organelle. Primary cilia are sensory, microtubule-based organelles that protrude from surface of cells and play a role in cell signaling. Precise measurement of the localizations of ciliary proteins has been the first and critical step of understanding their extraciliary functions in signaling. Hua and Ferland investigated this by measuring the localizations of 8 ciliary proteins using immunofluorescence based on both coagulative and cross-linking fixation protocols.^50^ Although the authors did not compare fixed-cell immunofluorescence to live-cell images, their work revealed major discrepancies between different fixation protocols. For example, fixation of the cilia marker protein adenylyl cyclase 3 (ADCY3) with a combination of 4% PFA and 4% sucrose and with 4% PFA diluted in cytoskeletal buffer yielded different results. While the PFA–sucrose method showed the marker localizing to cilia, the PFA–cytoskeletal buffer method instead revealed the presence of the marker in mitotic spindles. While they recommend fixation using 4% PFA diluted in cytoskeletal buffer or at least the use of cytoskeletal buffer as a general starting point, they conclude that there is no optimal fixation protocol among those they tested and that different fixation protocols may need to be used even for the same protein, depending on its subcellular localization of interest. The mechanism underlying the differential effects of the investigated fixation protocols is still unknown.

## Conclusion and Outlook

While applying fluorescence microscopy to fixed cells is a powerful way to study the subcellular localizations of proteins, care needs to be taken to ensure that the chosen fixation protocol faithfully preserves those protein localizations. Live-cell imaging of the protein under investigation after labeling it fluorescently, whether by genome editing to label an endogenous protein or by expressing an exogenous protein fused to a fluorescent tag, is often a helpful control to ensure that fixation is not introducing artifacts. Since there currently is no universal, artifact-free fixation protocol, it is often necessary to choose an appropriate fixation protocol on a case-by-case basis depending on the protein being studied. We discuss different mechanisms underlying fixation artifacts, including incomplete fixation causing the artifactual clustering of membrane receptors, coagulating fixatives deforming mitochondria, and slow fixation relative to the binding and unbinding dynamics of proteins associated with subcellular structures including chromatin and LLPS droplets. However, many other known fixation artifacts occur for yet unknown reasons, e.g., aldehyde- and organic solvent-based fixation protocols failing to preserve the localizations of ciliary proteins.

Our recent work suggests that a fast overall fixation rate relative to protein binding/unbinding rates can minimize the dynamics-dependent fixation artifacts.^1^ This idea is consistent with several earlier works. Notably, Teves et al. showed that high pressure freezing followed by freeze substitution, a standard fixation technique used for electron microscopy that slows down biomolecular motions, manages to partially preserve binding of Sox2 to mitotic chromatin, which is artificially released to the nucleoplasm upon PFA fixation.^2^ It will be of great future interest to develop novel fixation methods with significantly faster fixation rates than biomolecular interactions to eliminate many fixation artifacts in the cell.

## Abbreviations

LLPS: liquid–liquid phase separation
IF: immunofluorescence
PFA: paraformaldehyde
GA: glutaraldehyde
FA: formaldehyde
TF: transcription factor
HMG: high-mobility group
FRAP: fluorescence recovery after photobleaching
SPT: single-particle tracking
CTCF: CCCTC-binding factor
IDR: intrinsically disordered region
FISH: fluorescence *in situ* hybridization
Sox2: SRY-box transcription factor 2
Oct4: octamer-binding transcription factor 4
Esrrb: estrogen related receptor beta
Klf4: Krüppel-like factor 4
Sp1: specific protein 1
Foxo1: forkhead box protein O1
Foxo3a: forkhead box O3
GATA1: GATA binding protein 1
FoxA1: forkhead box protein A1
HNF1β: hepatocyte nuclear factor 1β
DSG: disuccinimidyl glutarate
FUS: fused in sarcoma
EWSR1: Ewing sarcoma breakpoint region 1/EWS RNA binding protein 1
TAF15: TATA-box binding protein associated factor 15
FTH1: ferritin heavy chain
MeCP2: methyl-CpG binding protein 2
TBP: TATA-binding protein
LYVE-1: lymphatic vessel endothelial hyaluronan receptor 1
CD31: cluster of differentiation 31
ER: endoplasmic reticulum
ADCY3: adenylyl cyclase
